# The effect of side chain engineering on conjugated polymers in organic electrochemical transistors for bioelectronic applications

**DOI:** 10.1039/d1tc05229b

**Published:** 2022-01-07

**Authors:** Yifei He, Nadzeya A. Kukhta, Adam Marks, Christine K. Luscombe

**Affiliations:** Materials Science and Engineering Department, University of Washington Seattle Washington 98195-2120 USA christine.luscombe@oist.jp; Department of Chemistry, University of Oxford Oxford OX1 3TA UK; Department of Chemistry, University of Washington, Seattle Washington 98195 USA

## Abstract

Bioelectronics focuses on the establishment of the connection between the ion-driven biosystems and readable electronic signals. Organic electrochemical transistors (OECTs) offer a viable solution for this task. Organic mixed ionic/electronic conductors (OMIECs) rest at the heart of OECTs. The balance between the ionic and electronic conductivities of OMIECs is closely connected to the OECT device performance. While modification of the OMIECs’ electronic properties is largely related to the development of conjugated scaffolds, properties such as ion permeability, solubility, flexibility, morphology, and sensitivity can be altered by side chain moieties. In this review, we uncover the influence of side chain molecular design on the properties and performance of OECTs. We summarise current understanding of OECT performance and focus specifically on the knowledge of ionic–electronic coupling, shedding light on the significance of side chain development of OMIECs. We show how the versatile synthetic toolbox of side chains can be successfully employed to tune OECT parameters *via* controlling the material properties. As the field continues to mature, more detailed investigations into the crucial role side chain engineering plays on the resultant OMIEC properties will allow for side chain alternatives to be developed and will ultimately lead to further enhancements within the field of OECT channel materials.

## Abstract 2

The versatile synthetic side chain toolbox assists in tuning the OECT parameters by controlling material properties of organic mixed conductors. In this review we critically summarise and evaluate various side chains used throughout OECT materials.

## Graphical abstract

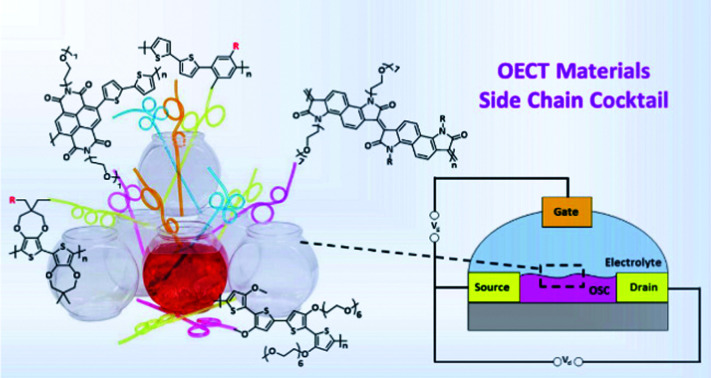

## 1. Introduction

It is difficult to imagine any underlying physiological process in living organisms without considering the role of ions. Ionic solutions in water and bodily fluids are major players in the regulation of essential biological and metabolic processes, as osmosis, pH monitoring, and hydration.^1^ Furthermore, ions are responsible for the stimulation and modulation of a plethora of crucial mechanisms in both animal (neural impulse, muscle function) and plant (turgor, photosynthesis) worlds.^2^ Any form of life is tightly interconnected with ionic behaviour.^3^ The nature and concentration of the latter can provide invaluable information about the health circumstances of a biological system.^4–7^ As such, qualification and quantification of ions, as well as the examination of any possible divergence from normality, underpins such applications as healthcare, environmental monitoring, biomedical diagnostics, security, and food/water quality control.^8–13^

**Fig. 1:**
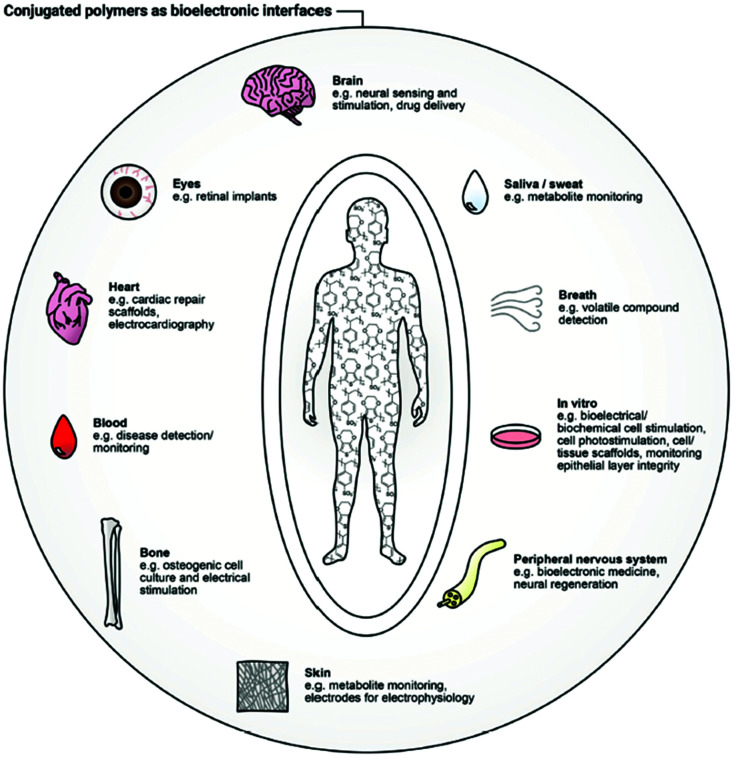
Bioelectronic interface with human body applications of conjugated polymers. Reproduced from ref. 16 with permission from Wiley-VCH.

To establish the origin of a complex biological condition or treat a disease, a responsive system capable of interacting with biological substrates and translating their characteristics into distinguishable electronic signals is necessary.^14^ Establishing the link between these biosystems and readable electronic output is a major focus of bioelectronics. Creating this connection is associated with a handful of difficulties, related to the fundamental differences in the operational modes and material features of human-made and nature-created structures.^15^ For instance, while biosystems tend to use ionic and molecular forms for information transfer, electrons and holes serve that role in artificial electronic systems. Typically, hydrophobic electronic devices are composed of rigid counterparts, while water-friendly biological systems are known to be flexible and soft. Diversity in energy sources and operational conditions conclude the list of differences. To address these divergences and merge them in an efficient bioelectronic device, the development of new materials, state-of-the-art device architectures, and appropriate power sources is essential.^15^ The result of this merging is a bioelectronic interface, capable of bidirectional recognition of biological signals (*e.g.*, cells, organs, tissues) induced by the change in electronic or ionic charge transport.^16^ Many applications have arisen as a result of the development of new bioelectronic interfaces: cell culture,^17^ biomedical diagnosis,^18^ electrophysiological stimulation^19^ to name but a few (Fig. 1).^20^ Inspired by the progress in other areas of organic electronics, namely organic field-effect transistors (OFETs), organic solar cells (OSCs) and organic light-emitting diodes (OLEDs), the field of bioelectronic devices has blossomed over the last two decades.^21^ Comparable to the famous pioneering Galvani's animal electricity^22^ experiment, advances in new bioelectronic materials lead to the device miniaturisation and sensitivity improvement.^23^

**Fig. 2:**
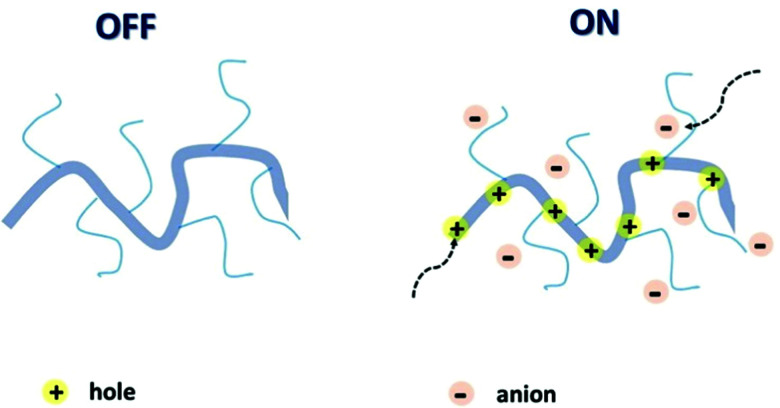
Schematic representation of an OMIEC acting as a channel material in an accumulation mode OECT. Reproduced from ref. 30 with permission from Springer Nature.

The attributes of secure and efficacious bioelectronic interfaces include biocompatibility, operational stability, compatibility with the living matter, sensitivity, and detection speed.^14^ All of these conditions can be met by an organic electrochemical transistor (OECT). High selectivity and sensitivity, signal transduction and amplification, permeability, and operational stability in plentiful biologically important electrolytes bring OECTs to the forefront of bioelectronic research.^24–26^ It is the ability of the polymer channel material to uptake ions and other metabolites from the interfacing electrolyte and transport electronic charge carriers (holes and/or electrons), resulting in mixed ionic and electronic conductance, that underpins the superior performance of OECTs in bioelectronic applications.^27^ Mixed conductance, permeability, and conformability, essential for the OECT operation, can be achieved *via* the utilisation of organic mixed ionic–electronic conductors (OMIECs).^28^ As opposed to conventional rigid inorganic electronics components, OMIECs possess the merits of facile low-temperature processability and solubility in various organic solvents, which makes them suitable for mass production printing techniques.^29^ These advantages stem from the distinctive molecular design of OMIECs, generally combining a highly conductive conjugated polymer (CP) backbone and side chains capable of ion uptake (Fig. 2).^30^

To fabricate a highly efficient OECT channel material, the condition of facile ion penetration^31^ through the CP network upon voltage application, has to be met.^32^ That provides significant transconductance values, which translate into the adequate sensitivity of target devices.^33^ CP's sensitivity to both ionic and electronic charge carriers furnishes them with suitability for a wide range of applications, using ion-to-electron signal conversion.^16,34^ Moreover, mechanical softness on par with that of biological tissues, has to be achieved for CPs to render them suitable for biomedical applications. The tuneability of CP underpins the versatility of OECT-based devices, and enables the wealth of applications.^16,35^ While modification of electronic properties is primarily dictated by the development of novel conjugated scaffolds, such properties as ion permeability, solubility, flexibility, morphology, and sensitivity can be tuned through side chain architectures.^36,37^ Even though the effects of side chains on the overall device performance have been studied extensively in other fields of organic electronics (*e.g.*, OFETs, OSCs, OLEDs),^38^ well-structured reviews on structure-performance side chain directed trends in OECTs are lacking. Therefore, the motivation behind this work is uncovering the influence of side chain molecular design of channel materials, on properties and performance of resultant OECT devices. Firstly, the fundamental concepts of the OECT physics and commonly used OMIEC materials will be discussed in Section 2, to summarise current understanding of OECT performance. Additionally, the concepts of the ionic–electronic coupling, sensitivity and selectivity will be introduced, and their connection with the side chain engineering approach will be uncovered. Further on, Section 3 will focus on the detailed discussion of OECT material side chains, showing how the versatile synthetic toolbox can be employed to tune various OECT parameters. Finally, an overview and perspective for future side chain development will be presented in the conclusion section.

## 2. Organic electrochemical transistors: fundamental concepts, bioelectronic applications and side chain engineering

### 2.1 OECT physics

**Fig. 3:**
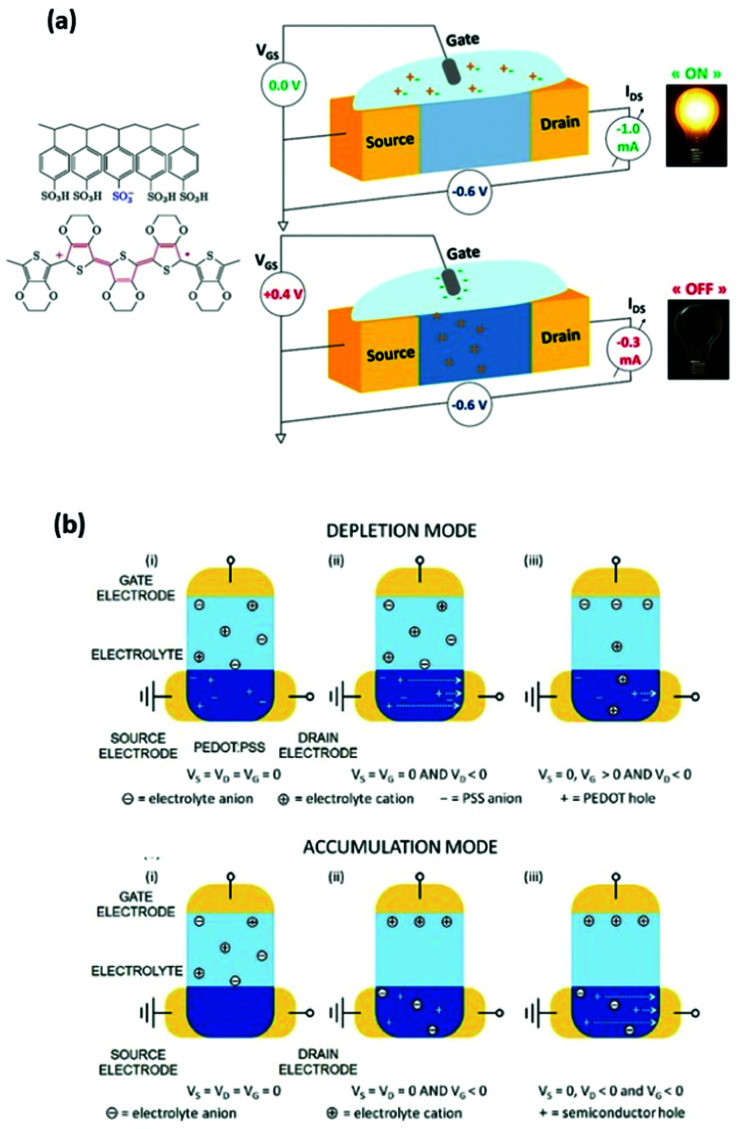
(a) Representation of the OECT operation principle, utilizing a typical OMIEC channel material, PEDOT:PSS. Reproduced from ref. 40 (b) top panel: depletion mode, where (i) unbiased state of a channel material (PEDOT:PSS); (ii) holes move towards the drain electrode upon positive gate voltage application; (iii) positive gate voltage causes the reduction of the hole flow. Bottom panel: accumulation mode, where (i) unbiased state of a channel material (p-type); (ii) negative gate voltage application causes electrochemical doping; (iii) holes move towards the drain electrode upon negative gate voltage application both at the gate and drain electrodes. Reproduced from ref. 21 with permission from Wiley-VCH.

The performance of an OECT is governed both by its device structure and the features of the materials involved. In parallel with other transistors, such as conventional OFETs and electrolyte-gated OFETs (EGOFETs), OECTs are miniature thin-film devices, comprised of a source, drain and gate electrodes, and a layer of channel material sandwiched between them (Fig. 3(a)).^21^ OECTs are known to operate in two modes, namely depletion and accumulation, depending on the nature of the channel material.^39^ A benchmark p-type channel material, namely poly(3,4-ethylenedioxythiophene) doped with polystyrene sulfonate (PEDOT:PSS) operates in the depletion mode (Fig. 3(b), (top)). Whilst the doped initial state of a channel material is a key characteristic of depletion mode OECTs, unbiased undoped CPs (p-type in this case) serve as a foundation for accumulation mode OECTs (Fig. 3(b), (bottom)). As the device switches on due to the hole build-up, both high hole mobility and superior neutral/oxidised state stability represent the crucial requirement for the accumulation mode p-type OECTs.^40^Fig. 3(b) details the processes of polymer doping in the cases of initially doped channel materials (PEDOT:PSS) and undoped p-type CPs. Electron mobility governed n-type CPs enable both depletion and accumulation modes of OECT operation. Needless to say, that stability requirement is equally applicable to n-type channel materials.^21^

The efficacy of the OECT performance can be described using transconductance (*g*_m_), which essentially defines the signal transduction by the transistor and dictates the OECT sensitivity. The scale of *g*_m_ largely depends on the OECT geometry and such material-specific characteristics, as charge carrier mobility and volumetric capacitance.^39,41^ The relationship between these parameters is outlined in eqn (1):1
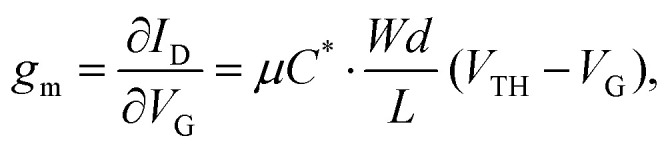
where *g*_m_ – transconductance, *I*_D_ – drain current, *V*_G_ – gate voltage, *μ* – electronic charge carrier mobility, C* – volumetric capacitance, *W* – channel width, *d* – channel depth, *L* – channel length and *V*_TH_ – the threshold voltage.

**Fig. 4:**
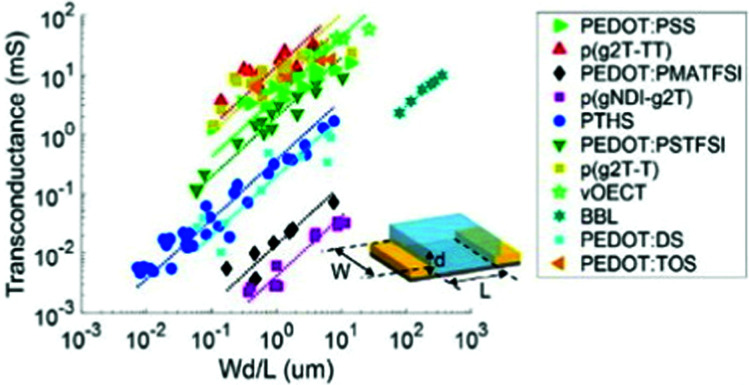
The ranges of transconductance values of various OMIEC materials. The dotted lines correspond to the best fits of transconductance *g*_m_ = *αWd*/*L*, with *α* being a proportionality constant.^42^ Reproduced from ref. 44.

Notably, OECT transconductance can exceed that of OFETs, reaching values as high as 800 S m^−1^.^42,43^ In bioelectronic applications, transconductance generally serves as a function of the parameter of interest (*e.g.*, target ion or metabolite concentration).^44^ Consequently, the high sensitivity of OECTs stems from enhanced resolution at reduced detection limits, which is accounted for by the gate voltage/channel current interconnection.^45^ The combination of such low operational voltage and high transconductance create beneficial conditions for the precise examination of biological events.^37^Fig. 4 presents the comparison of the transconductance values of a collection of the reported OMIEC materials.^44^

With the addressed significance of OECT transconductance in biological applications, as introduced above, enormous efforts have been made on the development of OECT channel materials, in which the signal amplification essentially occurs. The upcoming Section 2.2 presents an overview of currently well-studied OECT channel materials and introduces side chain engineering as a systematic tuning approach of material properties. The latter is further connected to the fundamental concept of ionic–electronic coupling through the discussion of morphology related effects (Section 2.2.1). Sensitivity and selectivity concepts will be discussed in detail further in Section 2.2.2, followed by the detailed examples of types of applied side chains in Section 3.

### 2.2. OECT materials and side chain engineering

The mode of operation of an OECT device is governed by the choice of channel material. Thus, an intrinsically doped material is expected to enable the depleted operational mode, while a CP requiring additional doping would warrant the accumulation mode. The OECT channel materials can be classified according to the type of organic semiconductor involved: p- or n-type.^46^ Additionally, OMIECs for use in OECTs can be subdivided following their chemical composition. Homogenous materials support bidirectional electronic and ionic charge transport either in a single material or in a materials blend, whilst heterogeneous type refers to the segregated regions of exclusively ionic or electronic transport.^15^

The work-horse OECT material, which has found numerous bioelectronic applications, is PEDOT:PSS. PEDOT:PSS is a p-type depletion mode channel material, whereby application of the positive potential at the gate electrode of an OECT leads to cation injection and, consequently, to PEDOT:PSS dedoping.^47–49^ However, being a heterogeneous system, PEDOT:PSS does not provide much freedom for synthetic modifications. Hence significant efforts were dedicated to the design of accumulation mode homogeneous CPs as potential improvements.^50^

p-Type CPs are mainly represented by thiophene-based materials. Polythiophenes are particularly attractive due to their overall (thermal, chemical, environmental) stability in both doped and undoped states. Even though unsubstituted polythiophene reveals some solubility issues, synthetic incorporation of side chains resolves this issue. For instance, decoration of the polythiophene scaffold with long alkyl chains and development of facile synthetic methods (*e.g.*, Kumada catalyst transfer polymerisation) has led to the introduction of the most archetypal polythiophene derivative, namely regioregular poly(3-hexylthiophene-2,5-diyl) (P3HT).

As electron mobility represents an obvious challenge for the p-type thiophene-based materials, a new class of n-type CPs began to emerge. Efficient n-type OMIECs have been prepared, utilising a donor–acceptor molecular skeleton bearing a strong electron accepting 1,4,5,8-tetracarboxylic acid diimide (NDI) fragment. Compared to PEDOT:PSS, significantly higher sensitivity and signal amplification could be achieved for n-type OMIECs.^40^ The combination of the NDI chromophore with the functionalised thiophene moiety resulted in low reduction/oxidation potentials of the OMIEC copolymer and enabled ambipolar p- and n-type OECT performance.

In addition to the synthetic progress in designing polymer backbones, the efforts on the side chain engineering offer another key aspect in developing future OMIECs. Side chain engineering allows further fine tuning of material properties, which brings ease to set up model studies in lab and ultimately helps establish our understanding of the structure–property relationship of OECTs, *e.g.*, the concept and determining factors of ionic–electronic coupling. In the next section, the impact of side chains on this electrochemical event will be addressed, which further demonstrates how side chain engineering would substantially influence the sensitivity of OECTs. More importantly, selectivity of OECTs with respect to the biological analytes also benefits from the incorporation of functional side chains, which will be discussed in Section 2.2.2 subsequently.

#### 2.2.1. Side chain engineering effect on ionic–electronic coupling

**Fig. 5:**
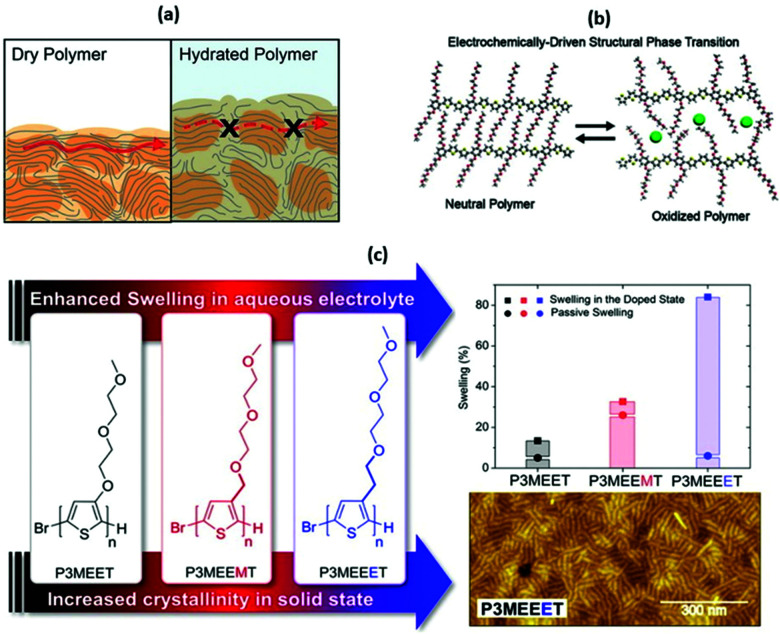
(a) A cartoon representing the differences in electronic charge transport in dry and hydrated and doped OMIEC materials. Reproduced from ref. 50 with permission from the American Chemical Society. (b) The role of side chains in the OMIEC doping mechanism. Reproduced from ref. 76 with permission from the American Chemical Society. (c) Chemical structures of no spacer, methyl spacer and ethyl spacer P3MEET derivatives, comparative swelling data and AFM image of P3MEEET. Reproduced from ref. 89 with permission from the American Chemical Society.

In the context of OECTs, not only do the optoelectronic properties of the materials matter, but also doping-related characteristics and consequent variations in ionic–electronic coupling. As a result, OECT mobility and transconductance values can be greatly affected by side chain modification. OECT sensitivity and selectivity can be directed by the choice and incorporation of appropriate side chains.^51^ Ionic–electronic coupling refers to the balance of electronic and ionic conduction within a mixed conducting material. Electronic conduction is governed by the equilibrium of charge concentration and charge mobility, which consequently depends on structural and morphological features. In terms of molecular design, efficient π-orbital overlap is crucial to enable electronic transport. Such an overlap can occur both within the chain of a conjugated polymer (intramolecularly) and upon the polymer chain through-space π–π interaction (intermolecularly). The combination of simultaneous inter- and intramolecular π-orbital overlap allows for efficient electronic transport along and between the polymer chains. Using rigid molecular fragments and assembling planarised scaffolds has proven to be a successful approach towards efficient conjugation, bandgap minimisation, and consequently enhanced electron conduction.^52,53^ Importantly, intermolecular interchain π–π interactions have a significant impact on polymer morphology *via* the emergence of crystalline regions, responsible for higher electronic transport (Fig. 5(a), left panel).^50,54,55^ Side chain engineering offers a wonderful tool to tailor electronic conduction within the conjugated polymers by means of fine-tuning the bandgap and affecting the π–π stacking of the polymer backbone.^38^ For example, controlling the side chain length and the degree of branching could significantly alter the π–π stacking distance and thus control the intermolecular charge hopping for the application of OFETs.^28,56,57^ This general rule is applicable to OECT channel materials in terms of improving electronic conductance, and has been translated from the well-established alkyl side chain system to the wide spreading ethylene glycol based side chain system, detailed later.

While the above observations apply to dry polymer films, the situation changes with the volumetric electrolyte uptake (Fig. 5(a), right panel).^50^ Upon exposure to the electrolyte solution, the CP film is subject to swelling. Swelling in turn promotes infiltration of the ions and water molecules into the bulk of the polymer film. As a result, initial polymer swelling constitutes an important condition of ionic conductivity.^52^ Ions are not only subject to hopping, but also follow the Grotthus mechanism of solvated ion transport, resulting in amplified ion conduction.^58,59^ Since most of the currently studied CP backbones are hydrophobic, side chain engineering serves another important role by introducing hydrophilicity into the system to promote effective ionic–electronic coupling.^51,60^

The extent of the ionic–electronic coupling greatly depends on the side chain segmental mobility, as well as side chain interaction with a dopant. In studies using the classical P3HT system, more efficient ion doping in the bulk thin film has been observed in the case of an amorphous P3HT when compared to the crystalline P3HT regions.^12,61–65^ The finding of heterogenous swelling demonstrates that the degree of water uptake within the polymer, upon OECT operation, is reliant on the polymer's crystallinity and microstructure, which could be tuned by different side chain engineering strategies reviewed later. Computational work from Dong and co-workers further suggest that a negligible change in the side chain design can significantly influence the morphology of the mixed conducting polymer *via* affecting the conformational order or the side chains in the amorphous domain, hence, modifying the conductivity values.^66^ Owing to their ionic–electronic coupling, mixed conductors in OECTs are capable of substantial current amplification in the presence of the analyte in question, which gives rise to satisfying sensitivity of OECT at a low operational voltage in the aqueous environment.^4,67^

#### 2.2.2 Side chain engineering effect on the selectivity of mixed conductors

On par with ionic–electronic coupling and the related sensitivity parameter as discussed above, selectivity represents another crucial parameter of the OECT bioelectronic device performance.^68^ Selective detection of certain biologically hazardous molecules in the presence of other analytes is important in healthcare applications.^69^ While the selectivity of an OECT is largely associated with the utilisation of ion-selective membranes^3^ and catalytic enzymes,^68^ CP side chains undeniably play a significant role in facilitating the selectivity by providing chemical linkage to various ligands and enzymes.

**Fig. 6:**
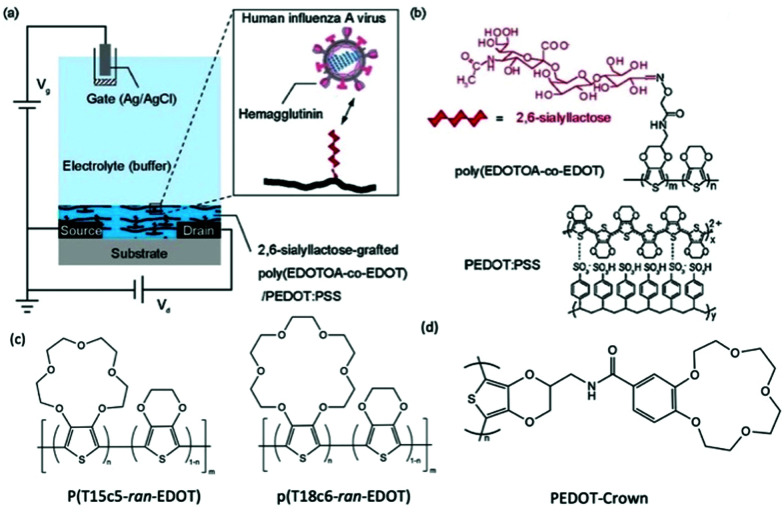
(a) Schematic of the OECT device, employing the poly(EDOTOA-*co*-EDOT)/PEDOT:PSS composite as the active channel material. (b) Chemical structure of the 2,6-sialyllactose functionalized EDOT derivative. Reproduced from ref. 72 with permission from Elsevier. (c) Chemical structures of crown ether functionalized PEDOT copolymer.^73^ (d) Chemical structure of PEDOT-Crown.^74^

Post-functionalisation of the channel materials has been prevailing to immobilise probing molecules on the material surface, due to the smaller synthetic barrier compared to pre-functionalisation strategy. However, the potential damage to the engineered molecules and device performance after common processing techniques, *e.g.*, plasma treatment, thermal annealing, and solvent erosion, brings trouble to efficient device fabrication.^70^ Strategies that avoid the above harsh post-processing conditions have to adapt the weak intermolecular forces, which limits the long term functionality of the device, as well as the grafting efficiency of biomolecules.^71^ Applying side chains as chemical linkages to the complicated biomolecules is currently an effective solution to this issue. The smaller size of chemical linkage as monomer side chain allows ease of polymerisation of the backbone, while offering strong connecting sites for the grafting of biomolecules which are later covalently bonded to the CP channel. In 2018, Hai *et al.*, presented a functionalised PEDOT:PSS derivative for human influenza virus sensing.^72^ More specifically, the authors covalently grafted 2,6-sialyllactose (an influenza virus receptor) onto an oxylamine moiety which was tethered to an EDOT-based backbone (Fig. 6). The OECT device was utilised as an effective signal transducer, whereby the binding interaction between 2,6-sialyllactose and hemagglutinin led to recordable fluctuations in the drain current. Moreover, the overall negative charge of the influenza virus nanoparticle incurred an anionic doping effect within the active channel, subsequently altering the drain current output of the OECT. Compared to common immunochromatographic tests, the poly(EDOTOA-*co*-EDOT)/PEDOT:PSS composite-based OECT devices demonstrated a two order of magnitude decrease in the limit of detection. Despite this, the devices are a low power consumption alternative, offering facile processing from printed technologies for mass production. Similarly, Galán *et al.*, report a selective sensor of Hepatitis C virus using DNA sequence functionalised PEDOT, which has been engineered with azide side chains first to serve as linkage to the virus probe.^71^ These studies highlight the versatility of side chain engineering in combination with the signal amplifying and transduction potential of an OECT device. The ability to bind specific biomolecules paves the way for wearable sensors and point-of-care evaluation of interested biological substances or events. The ability to synthetically tune the linker moiety also represents the potential to expand this design to target multiple other viruses.

The selectivity of OECTs could be further established by side chain engineering the gate electrode with crown ether functional groups. The size of the cavity in the crown ether units determines the specific alkali metal cations that would induce the intercalation effect. Such selective complexing between the targeted metal ions and the crown ether components causes the disruption of the π conjugation along the CP backbone, thus generating the reduction current that primarily correlates to the change of concentration of interested ions in the environment. Based on this fundamental mechanism, Wustoni *et al.*, copolymerised a traditional EDOT unit with crown ether engineered EDOT units as the coating of gate electrodes of OECTs for selective recognition of K^+^ and Na^+^. The crown ether functionalised PEDOT system allows selective sensing of the targeted cations in the physiological concentration range without any additional membrane filters as shown in Fig. 6(c).^73^ Additionally, Kousseff *et al.*, reported that functionalising crown ethers to the PEDOT system provides the material with better electrochemical stability and substrate adhesion, in addition to the metal cation selectivity.^74^

Summarising the above discussion of the side chain effects on OECT channel materials in biological applications, it can be concluded that the side chains have a direct influence on the following parameters of a mixed conductor: (i) HOMO/LUMO energy levels, affecting, in turn, the bandgap, linear electronic charge transport, and ionic–electronic coupling; (ii) π–π stacking of the polymer backbones, influencing through-space electronic charge transport and crystallinity; (iii) the extent of swelling and ion uptake, controlling the resulting morphology; (iv) sensitivity and transconductance of the resulting OECT; (v) the selectivity of the materials. In Section 3, we will review the different types of side chains which have been employed in OECT active channel materials, commenting on various device and material improvements imparted from the plethora of side chain engineering literature.

## 3. Types of side chains utilised for OECT active channel materials

In addition to the above fundamental mechanisms that give rise to the high sensitivity and selectivity of OECTs, uncovered by side chain related studies, side chain engineering has been extensively studied to provide a set of systematic tuning strategies for high performance OECT channel materials as well. Section 3 thus focuses on the selection of currently most studied side chain types, including the ethylene glycol family, the alkyl and alkoxy side chains, the hybrid side chains and finally the charged side chains. With various examined side chain parameters here, further detailed design principles will be revealed in this section.

### 3.1 Ethylene glycol (EG) based side chains

As discussed in Section 2, ionic conductance heavily relies on the hydrophilicity of CPs to facilitate ion flow inside the thin film. Given the difficulty of altering the hydrophobic backbones for most of the CPs, hydrophilic side chains have become an efficient solution. Currently, one of the most widely studied hydrophilic side chains is ethylene glycol (EG) based side chains. CPs bearing EG side chains are able to facilitate aqueous solubility, more aqueous ion transport, and the stabilisation of ions in the materials.^51,75^ It is reported that doping kinetics of the glycolated polythiophenes with respect to small anions could be approximately 150 times faster than their alkylated counterparts, implying its potential in biosensing applications.^50^

In addition to the facilitated water and electrolyte uptake, hydration brought by the introduced hydrophilic side chains induces morphological changes that allow the mechanism of charge injection. Bischak *et al.*, has recently conducted a detailed experimental study, uncovering the reversible structural phase transitions in the thiophene-based systems engineered with hydrophilic EG side chains.^76^ Upon ion injection and electrochemical oxidation, the primary morphology of the glycolated mixed conductor poly[2,5-bis(thiophenyl)-1,4-bis(2-(2-(2-methoxyethoxy)ethoxy)-ethoxy)benzene] (PB2T-TEG) is commanded by the side chain-induced crystallisation. The cumulative effect of the hydration and injection of the ions facilitates the unzipping of the intertwined polymer chains. The latter is subject to the subsequent π–π-stacking governed zipping upon the oxidation (Fig. 5(b)).^76^ Such controllable phase transitions were advocated to be effective to tailor the electrochemical characteristics of mixed conductors. Significantly, the above-described phase transitions are dependent on the hydrophilic nature of the EG side chains, as no zipping/unzipping associated charge injection was observed in the alkylated P3HT polymer in the same study. With all these benefits, the current highest μC* reported for a p-type OMIEC reaches 522 F cm^−1^ V^−1^ s^−1^.^77^ However, from the published literature, it is obvious that the OECT performance of the tuned materials do not simply scale with the addition of EG side chains. Careful consideration is required to explore in detail how the introduction of hydrophilic side chains alter the performance.

**Fig. 7:**
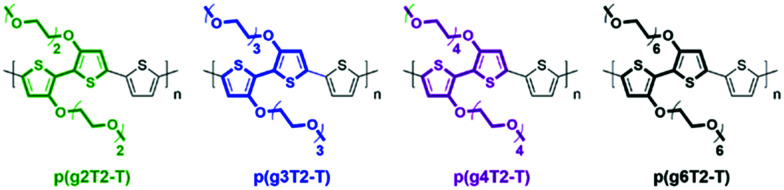
Chemical structures of p(g2T2-T), p(g3T2-T), p(g4T2-T), and p(g6T2-T). Reproduced from ref. 46 with permission from the American Chemical Society.

Modifications of EG based side chains have been manipulated using different parameters, including the overall chain length, the linkage spacer to the backbone, the total percentage in the bulk material, and finally the engineered backbone positions. As previously studied in the alkylated system, the side chain length could systematically tune the morphology and thus the electronic conductance.^56^ An analogous study on the length of EG side chains was performed by Moser *et al.*, increasing the repeating units of the ethylene glycol side chains, tethered to a thiophene backbone, from 2 to 6 as shown in Fig. 7.^46^ Among the four presented glycolated polythiophenes, p(g3T2-T) exhibited the optimised volumetric capacitance and charge mobility, rendering an overall μC* exceeding 135 F cm^−1^ V^−1^ s^−1^. Increasing EG side chain length from p(g3T2-T) to p(g4T2-T) significantly decreases the charge mobility from 0.16 to 0.06 cm^2^ V^−1^ s^−1^, adding flexibility to the polymer backbone, which impedes long range ordered packing in a large range. On the other hand, p(g3T2-T) also achieves the optimised point of volumetric capacitance, within the series, with a value of 211 ± 18 F cm^−1^, due to the sufficient ion transport and stabilisation provided by the TEG side chains. Further addition of EG repeating units beyond three did not provide additional ion stabilisation, instead resulting in a decreased capacitance. As for the comparison with samples having decreased side chain length, p(g2T2-T) bearing the shortest EG side chains shows difficulty in processing into the OECT channel and efficiently transporting ions, due to the reduced solubility and disordered morphology.

**Fig. 8:**
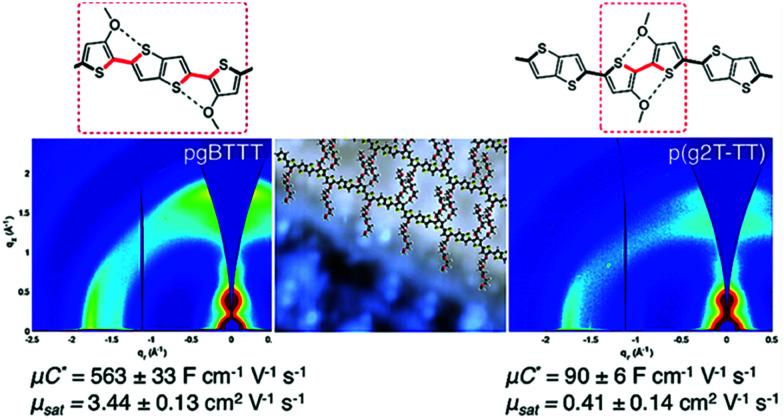
Sections of chemical structures of pgBTTT and p(g2T-TT) addressing S–O interactions with corresponding GIWAXS maps. Reproduced from ref. 78 with permission from the American Chemical Society.

In addition to the side chain length, researchers also investigated the effect of EG side chain position on the polymer backbone. Specifically, Hallani *et al.*, has reported that moving the 3′3 glycol side chains to the 4′4 position on the poly(2-(3,3′-bis(2-(2-(2-methoxyethoxy)ethoxy)ethoxy)-[2,2′-bithiophen]-5-yl)thieno[3,2-*b*]thiophene) (p(g2T-TT)) backbone significantly promotes the planarity and the alignment, resulting in an impressive hole mobility of 3.44 ± 0.13 cm^2^ V^−1^ s^−1^ and hence μC* of 502 ± 18 F cm^−1^ V^−1^ s^−1^.^78^ The partial chemical structures of the synthesised polymers are represented in Fig. 8. The resultant polymer, poly(2-(4,4′-bis(2-methoxyethoxy)-5′-methyl-[2,2′bithiophen]-5-yl)-5-methylthieno[3,2-*b*]thiophene) (pgBTTT), has a comparable degree of passive swelling with respect to p(g2T-TT), with the former one being 16 wt% and the other 12 wt%, respectively. However, upon the application of the doping potential, the active swelling differs significantly between these two polymers, with pgBTTT expanding by 46 wt% and p(g2T-TT) by 65 wt%. The reduced extent of active swelling in pgBTTT lowers its volumetric capacitance, whereas introducing much less disruption of the crystalline regions, which ensures high hole mobility that compensates for the lowered capacitance and still leads to a high value.

**Fig. 9:**
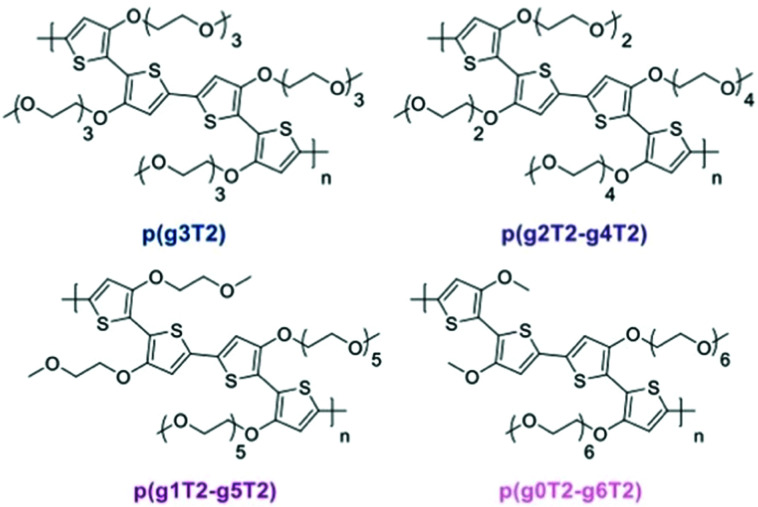
Ethylene glycolated polythiophenes with ‘redistributed’ side chain lengths. Reproduced from ref. 77.

Redistribution of EG side chains also impacts the electrochemical performances of CPs.^77^ By redistribution, Moser *et al.*, varied the number of repeating units of the EG side chains attached on neighbouring thiophenes while keeping the total amount of units the same, as shown in Fig. 9. Among the investigated polymers, p(g2T2-g4T2) shows the best μC* up to 522 F V^−1^ cm^−1^ s^−1^ followed by p(g1T2-g5T2) with μC* of 496 F V^−1^ cm^−1^ s^−1^. The important parameters of the remaining polymer samples are compared in Table 1.

**Table 1:** The electrochemical performances and swelling degree of a series of ethylene glycolated polythiophenes with ‘redistributed’ side chain lengths^77^

Polymer	*d* [nm]	*g*_m_ [mS]	C* [F cm^−3^]	*μ*_OECT_ [cm^2^ V^−1^ s^−1^]	[μC*] [F V^−1^ cm^−1^ s^−1^]	Active swelling [wt%]
p(g3T2)	75	8.9 ± 1.0	156 ± 1	0.90 ± 0.10	161	249
p(g2T2-g4T2)	45	6.5 ± 1.6	187 ± 8	1.72 ± 0.31	522	168
p(g1T2-g5T2)	65	10.2 ± 1.2	133 ± 3	2.61 ± 0.30	496	10
p(g0T2-g6T2)	70	8.1 ± 1.0	74 ± 4	2.95 ± 0.37	302	4

It is worth noting that the change of *μ* and C*, respectively, under this side chain manipulation also reveals a trade-off between electronic conductance and the ionic conductance as reported in the adjustment of the side chain length.^46^ With more data regarding the active swelling of the material in this study, evaluation of how these two parameters are related to the extent of expansion can be elucidated. As summarised in Table 1, *μ* is inversely correlated and C* is positively correlated to the degree of active swelling. Intuitively, increasing the hydrophilic chain length resulted in an increase of ion and water uptake and thus the active swelling; however, this relationship becomes weaker when the number of the repeating units exceeds 3, which is in agreement with the previous chain length study.^46^ Hence, the extent of active swelling is related more to monomers having the side chain length below 3 units, which explains the increased active expansion of the materials when the shortest side chain length is increased from 0 to 3 units. As the ability of water and ion uptake increases, the charge carrier mobility consequently decreases, which could be attributed to the further disruption of the crystalline regions in the materials. On the other hand, the volumetric capacitance, the metric that closely depends on the material's ability to transport and stabilise ions during doping, increases with the degree of active swelling. A slight decrease occurred for p(g3T2) since its additional expansion results from water uptake rather than ion transport into the bulk material. This study again addresses the importance of the trade-off between *μ* and C* or the future molecular design of OECT channel materials. Additionally, the stability of the OECT materials in aqueous environment has been significantly improved, with only 2% reduction of the initial channel current after 700 doping cycles for p(g3T2), with the previous benchmark glycolated polymer p(g2T-TT) suffering a 25% decrease of the initial current under the same testing conditions.^51^

**Fig. 10:**
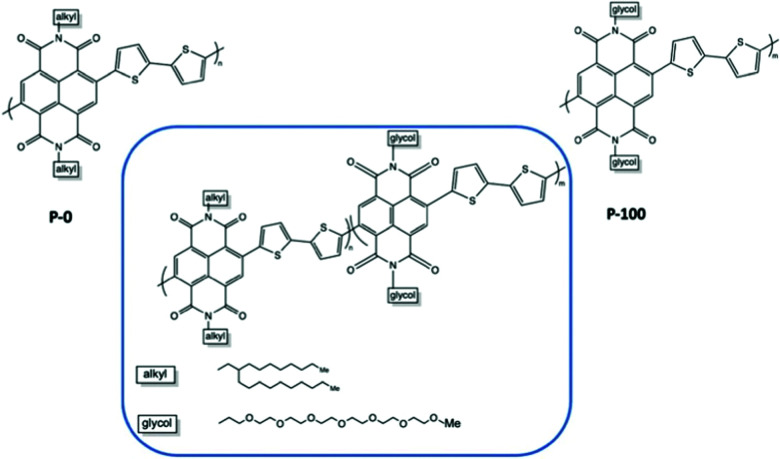
Chemical structures of the investigated mixed conducting copolymers, the P0 to P100 series.^37^

The percentage of EG chains is another dimension to look into when tuning the electrochemical performances of the material.^37,79^ Giovannitti *et al.*, gradually replaced alkyl side chains by ethylene glycol side chains in random copolymers based on naphthalene-1,4,5,8-tetracarboxylic-diimide-bithiophene (NDI-T2) as shown in Fig. 10.^37^ With the increasing percentage of glycolated monomers, the material starts to exhibit a dominant OECT operation mode, with diminished OFET performance, when the EG percentage exceeds 50%, which is aligned with previous research.^51^ As summarised in Table 2, when the primary working mode of the material transitions from OFET to OECT, the charge mobility drops drastically. Such decrease of efficiency in charge transport is highly related to the morphological changes brought by the addition of EG side chains. Analysing the morphology changes reveals that the lamellar spacing inside the film increases with the amount of glycol side chains. Addition of EG side chain fraction results in a stronger tendency towards disordered microstructures, regardless of water presence. Needless to say, the increased swelling of materials with increasing glycol side chain fraction further interrupts the interconnectivity among crystallites.

**Table 2:** The electrochemical performances and swelling degrees of NDI-T2 copolymers with increased EG side chain percentage from 0% to 100%^37^

Polymer	C* [F cm^−3^]	*μ*_OFET_ [cm^2^ V^−1^ s^−1^]	*μ*_OECT_ [10^−4^ cm^2^ V^−1^ s^−1^]	[*μC*] [10^−4^ F V^−1^ cm^−1^ s^−1^]	Swelling
P-0	—	0.132	—	—	<10%
P-10	—	0.0514	—	—	<10%
P-25	—	0.00184	—	—	<10%
P-50	—	—	—	—	<10%
P-75	188.0	—	1.46	274	12%
P-90	198.2	—	2.38	472	42%
P-100	192.4	—	1.96	377	102%

**Fig. 11:**
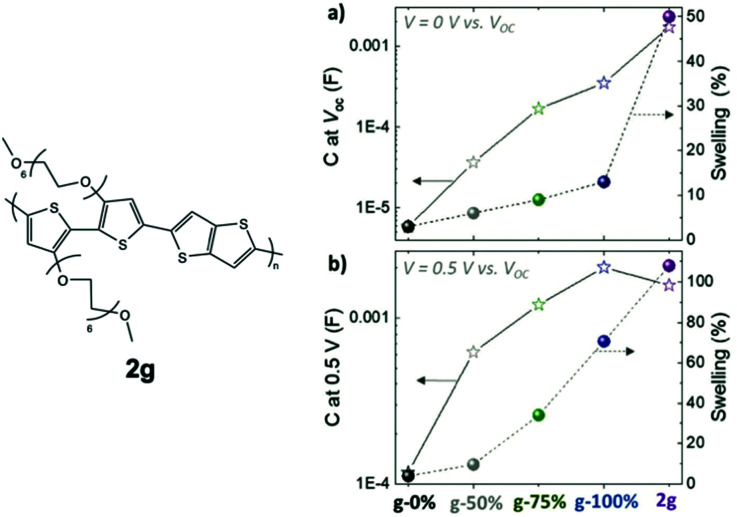
The swelling behaviour and volumetric capacitance with respect to different glycol fractions in p(g2T-TT) and 2g based polymers at (a) *V* = 0 V and (b) *V* = 0.5 V *versus V*_OC_. Reproduced from ref. 79 with permission from Wiley-VCH.

A similar study examining the impact of the EG side chain percentage on p-type conjugated polymers was summarised here for comparison, which provides more details regarding the trend between the TEG side chain fraction and the material properties.^79^ In this study, the portion of glycolated monomers was gradually increased in p(g2T-TT) copolymers from 0% to 100%, with an additional homopolymer bearing hexakis-EG side chains, named as 2g in Fig. 11. It is observed that the transconductance of the material scales by five orders of magnitude with the amount of EG side chains. As indicated in Table 3, such enhancement in *g*_m_ results from an increase in volumetric capacitance and a trend of *V*_TH_ to approach 0 V. The study also revealed that a higher portion of EG contents comes with greater swelling, which couples with improved volumetric capacitance with or without the electrochemical potential, as shown in Fig. 11. Characterisation of the properties and morphological changes occurred in 2g implies essential lessons of controlled hydrophilicity in the system. The volumetric capacitance of 2g upon the application of electrochemical potential drops slightly compared to g-100%. Uncoupling the doping process of anions and water molecules reveals that ion uptake reaches saturation with the amount of hydrophilicity in g-100%. Additional volume expansion occurred in 2g is a consequence of excessive water uptake, which indeed dilutes the electronic contents and lowers C*. In addition, with further penetration of water molecules, 2g films possessing the highest EG content shows the most severe heterogenicity in the swelling of crystalline and amorphous regions, with greater expansion of amorphous regions being observed, which sets the barrier for charge transport between crystallites. As a result, the hole mobility undergoes a steep drop.

**Table 3:** The electrochemical performances of p(g2T-TT) polymers with increasing amount of glycol side chains from 0% to 100%.^79^

Polymer	*g*_m_ [mS]	C* [F cm^−3^]	*μ*_OECT_ [cm^2^ V^−1^ s^−1^]	[μC*] [F V^−1^ cm^−1^ s^−1^]	*V*_TH_ [V]
g-0%	6.9 × 10^−5^	19	5.2 × 10^−5^	0.000988	−0.39
g-50%	0.048	97	0.009	0.873	−0.27
g-75%	7.1	206	0.38	78.3	−0.21
g-100%	18.8	297	0.55	163	−0.08
2g	1.3	231	0.07	16.2	−0.08

**Fig. 12:**
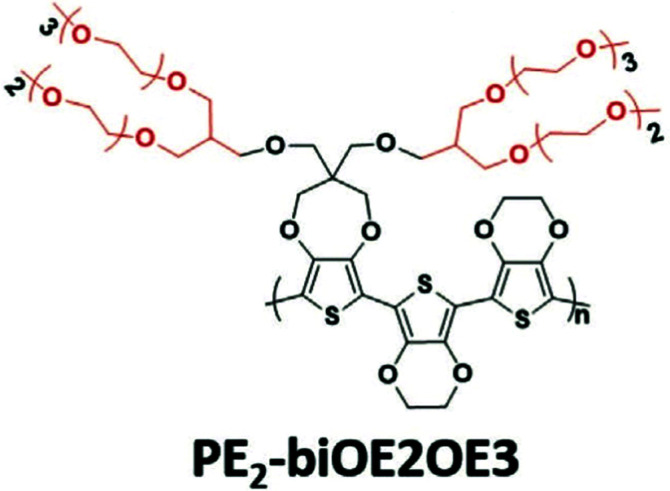
The chemical structures of reported green solvent processable PE_2_-biOE2OE3. Reproduced from ref. 82 with permission from Wiley-VCH.

A weakness of conjugated polymers incorporating a great amount of ethylene glycol side chains is their poor solubility in benign organic solvents, so that their processing has to involve toxic organic solutions such as chlorinated solvents.^37,75,80,81^ Introducing branching is an efficient solution to alleviate the processing conditions of these materials.^82,83^ Jones *et al.*, has recently reported an acetone-processable CP, PE_2_-biOE2OE3, that could be readily applied as a OECT channel material, with the chemical structure shown in Fig. 12.^82^ Upon the electrochemical potential, the loss of the initial channel current is only 10% after 1000 cycles. Similarly, Kang *et al.*, exploit the benefit of branched EG side chains and synthesised a series of water/ethanol processable CPs with a high hole mobility of 0.1 cm^2^ V^−1^ s^−1^ in the dry state. Although the latter study didn’t characterise the materials with OECT performance, branching of EG side chains could still be a step towards the fabrication of green OECTs in the future.^83^

Introduction of a crown ether, as a special case of EG side chains, represents an elegant way to fine tune the morphology, adhesion and electrochromic behaviour of the CPs (Fig. 6(c) and (d)). Upon decorating PEDOT with a crown ether (PEDOT-Crown) (Fig. 6(d)), Kousseff *et al.*, was able to achieve a superior material performance in electrochromic devices than that of the parent PEDOT.^74^ More advantageous properties of PEDOT-Crown were attributed to the improved surface morphology, stronger adhesion to ITO, more pronounced faradaic behaviour, and bathochromically shifted absorption features. Furthermore, the cyclic nature of the crown ether side chains was found to show selectivity toward alkali metal ions, suggesting improved suitability for biological sensing application as already discussed in detail in Section 2.2.2.^73,74^

The incorporation of hydrophilic EG side chains has been one of the most achieved side chain engineering strategies for competitive OECT channel materials, as proven by the significantly improved μC* and thus the transconductance values reported above. Given the numerous control studies on different dimensions of EG side chains, using side chains to tune the morphological response of channel materials upon the stress of electrochemical voltage has provided a more detailed guidance of material design. Specifically, the hydrophilicity should be introduced with care to reach both the sufficient ion uptake and minimal microstructure disruption. To further explore how the side chain nature would control the OECT performance, the role of hydrophobic side chains is later summarised to complement our understanding.

### 3.2 Alkyl and alkoxy side chains

With the great leap in bringing up transconductance values from the perspective of increasing hydrophilicity of the system, as presented in the previous section, the negative impacts inflicted by the repetitive swelling behaviour becomes clear as well. Lessons from the above studies indicate the necessity to control excessive water uptake beyond an optimised point, which not only accommodates less ion transport but also disrupts the ordered microstructure more severely. Moreover, the accumulation of the remaining water in each doping/dedoping cycle, due to the strong interaction between the glycol side chains and water molecules, leads to irreversible morphological changes that sets limits on the reversibility, stability and finally the performance of materials.^84,85^

**Fig. 13:**
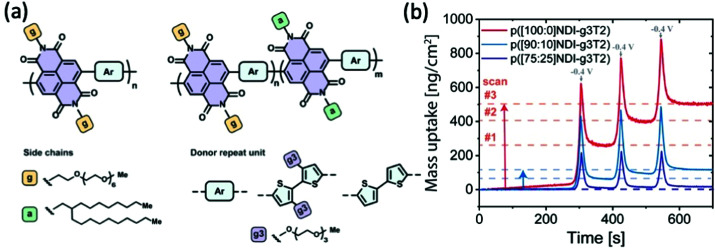
(a) The chemical structures of D–A type p(NDI-T2) and p(NDI-g3T2) with alternating hydrophilic and hydrophobic side chains on the acceptor monomers. (b) The mass uptake changes of the series of NDI polymers with the increasing percentage of hydrophobic alkyl side chains. Reproduced from ref. 84 with permission from the American Chemical Society.

In addition to increasing the backbone rigidity of the materials to improve the structural stability upon cyclic hydration, tuning the fraction of hydrophobicity becomes a solution toward this issue. Szumska and co-workers have reported an NDI based D–A type copolymer suitable for the electrochemical application in the aqueous environment, with the NDI acceptor monomers bearing alternating hydrophilic EG side chains and hydrophobic alkyl side chains, as shown in Fig. 13(a).^84^ This study examines the impact of side chain nature in a similar way to the EG percentage study previously discussed,^37^ but with a different focus on the role of alkyl side chains to control the swelling behaviour. Regardless of the slight difference in the donor units, the increasing amount of hydrophobic alkyl side chains results in a decrease in the degree of swelling in both types of polymers. The restricted swelling brought higher stability of the overall materials under the cyclic electrochemical voltages due to less irreversible disruption of the microstructures. Moreover, the retention of water is significantly controlled as represented by the measurement of the mass change of materials in each cycle shown in Fig. 13(b). The baseline of the polymer bearing purely glycolated NDI units drifted significantly in only 3 cycles of scans, whereas the baseline of polymers involving alkyl side chains have more stable baselines throughout the assessment. It is also noteworthy that controlled water uptake by the introduction of hydrophobic alkyl chains allows a higher fraction of utilisation of theoretical capacity in the material. Thus, the importance of tuning the ratio of hydrophobicity and hydrophilicity in the bulk material is thus revealed.

**Fig. 14:**
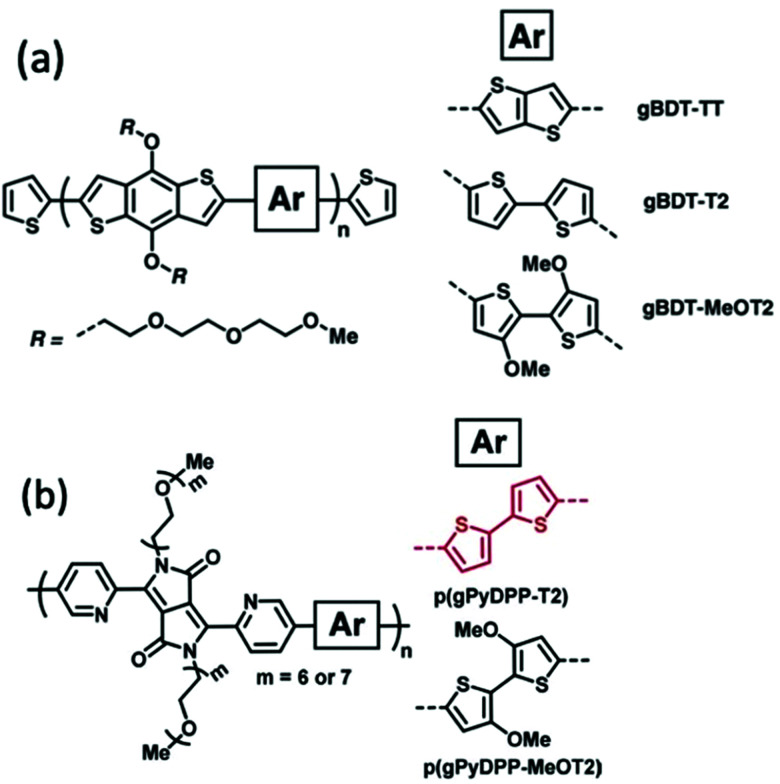
(a) The chemical structures of gBDT-TT, gBDT-T2, gBDT-MeOT2. (b) The chemical structures of p(gPyDPP-T2) and p(gPyDPP-MeOT2). Reproduced from ref. 41 and 86 with permission from Wiley-VCH.

Alkoxy side chains help to improve the electrochemical stability of CPs in a different way from alkyl side chains. This group of electron rich side chains could tune the ionisation potentials and thus the stability of conjugated polymers for application in OECT devices.^41,86^ In 2018, Giovannitti *et al.*, reported the improvement in the redox stability of benzo[1,2-*b*:4,5-*b*′]dithiophene (BDT) structure based D–A copolymers, *via* the choice of the comonomers, specifically the side chain difference.^86^ Among the series of glycolated BDT copolymers synthesised with comonomers bearing different side chains, as shown in Fig. 14(a), gBDT-MeOT2 exhibited a significant improvement in material stability and transconductance when applied as the channel in OECTs. With the introduction of oxygen atoms along the side chains of the comonomers, the oxidation potential of the overall material is lowered, thus requiring lower turn on voltage, preferable in a biological application. In addition, gBDT-MeOT2 shows improved solubility in common organic solvents compared to the rest of the copolymers. A follow up study from Giovannitti *et al.*, regarding the impact of alkoxy side chains engineered on the donor comonomers was subsequently conducted.^41^ In this series of synthesised copolymers, presented in Fig. 14(b), p(gPyDPP-MeOT2) exhibited greater redox stability with almost no loss of the initial current after 400 cycles at a gate voltage of −0.5 V, compared to its unsubstituted analogue. The higher stability could be credited to the greater extent of hole polaron localisation provided by the methoxy side chains. Moreover, the electron rich methoxy groups in the donor units help shield the polymer backbone from undesired reactions with oxygens when applied in the ambient environment.

From the studies discussed above, it can be seen that D–A type comonomers allow more flexibility in side chain engineering design, in terms of tuning the portions of mixed types of side chains to achieve the improved material properties. In both donor and acceptor units, the side chain engineering contributes significantly to the controlled polymer swelling and the increased stability.

Nevertheless, there lacks a comprehensive study that enhances both the performance metrics and the stability *via* blending mixed types of side chains in copolymers. Although the reported CPs in this section have less competitive performance compared to the current state-of-art materials,^41,86^ the ability of alkyl and alkoxy side chains to finely control the material swelling and stability addresses comprehensive consideration of impacts from both the hydrophobicity and the hydrophilicity constituents in the system.

### 3.3 Hybrid and ‘spacer’ side chains

**Fig. 15:**
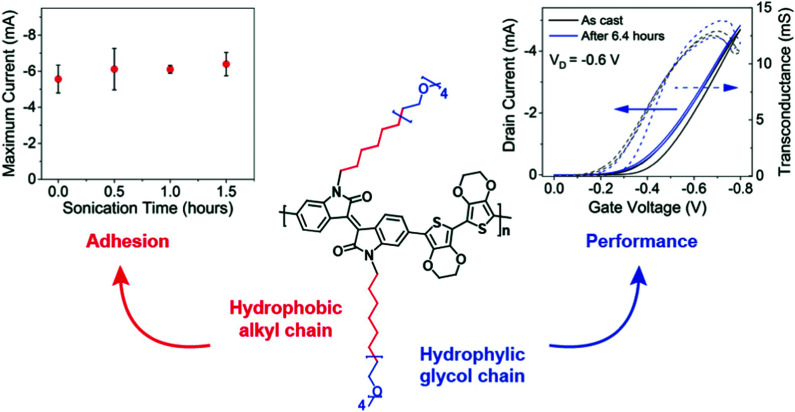
Maximum current *versus* sonication time (left), OECT transfer curve (right) and structure of PIBET-AO (middle). Reproduced from ref. 80 with permission from the American Chemical Society.

Given the above consideration, hybrid side chains become another side chain category that has been rigorously investigated. It has often been the case that a combination of these two types of side chains is required to impart solubility/processability (hydrophobic) and to facilitate conduction of aqueous ionic species (hydrophilic, most commonly EG based side chains) of the resultant polymer, hence enhancing the ionic–electronic coupling as discussed in Section 2.2.1. In addition to tethering side chain moieties of different natures to different comonomer, as discussed in the examples of Sections 3.3.1 and 3.3.2,^37,87^ an alternative design strategy was presented by Yue *et al.*, whereby a hybrid side chain, combining a hydrophobic alkyl component with a hydrophilic EG unit within the same side chain, was attached to an isoindigo backbone and polymerised with a bis(3,4-ethylenedioxythiophene) (bis-EDOT) donor unit, affording PIBET-AO (Fig. 15).^80^ The authors hypothesised that merging these components into a single side chain would retain the polarity necessary to induce ionic conduction (hydrophilic EG unit) whilst simultaneously preventing film dissolution (hydrophobic alkyl unit). In order to compare the effects on both OECT performance and polymer microstructure four different side chain compositions were investigated, namely: the hybrid mixed alkyl-EG side chain (PIBET-AO), linear and branched EG side chains, and branched alkyl side chains.^80^

The hybrid alkyl-glycol side chain substantially increased polymer to substrate adhesion, preserving operation and performance after 90 minutes of ultrasonication in an aqueous electrolyte (Fig. 15). In contrast replacing the hybrid side chain with a linear glycol side chain led to complete film delamination terminating device operation, over the same 90 minute time period. Notably, the hybrid alkyl-EG side chain decorated polymer displayed impressive operational stability, with OECT devices retaining their original current over a six-hour period of on–off cycling, totalling 3628 cycles. In contrast, the glycolated derivative (PIBET-O), whereby the hybrid side chains were replaced by linear 6-unit EG chains, retained only 10% of the original current after only 400 cycles. Upon increasing the hydrophilic side chain density, by incorporating a branched EG side chain (PIBET-BO), the operational stability decreased again with a 90% reduction in initial ON current after only 6 minutes (20 cycles). The poorer operational stabilities, moving from hybrid alkyl-EG to linear EG to branched EG, were prescribed to the increase in threshold voltage (and thus the voltage required to switch on the device) leading to overoxidation and increased swelling. These findings were corroborated by a recent study which demonstrated the importance of side chain composition on the resultant electrochemical stability, with a balance of hydrophilic and hydrophobic side chains increasing the redox reversibility of OMIECs in aqueous environments.^84^

A second study investigated the effect of introducing the methyl spacer by comparing P3MEEMT to an analogue containing an identical EG side chain directly tethered to the thiophene backbone (P3MEET).^88^ A combination of computational and experimental chemistry was employed to probe ionic transport properties and the resultant ionic conductivity of the two materials. A follow-up study expanded the derivatives to three, introducing an ethyl spacer between the thiophene backbone and the EG side chain to afford P3MEEET (Fig. 5(c)).^89^ The three homopolymers were investigated in OECTs and showed drastic variation in performance metrics, highlighting the importance of precise side chain engineering for improved performance as an active OECT channel material.^89^ The systematic increase in the alkyl linkage between the thiophene backbone and the EG side chain lead to an increase in volumetric capacitance from 80 ± 9 to 242 ± 17 F cm^−1^ between P3MEET and P3MEEET. Furthermore, moving from no spacer to methyl and finally ethyl spacers resulted in the OECT figure of merit μC* value to increase by more than two orders of magnitude, from 0.04 ± 0.01 to 11.5 ± 1.4 F cm^−1^ V^−1^ s^−1^, for P3MEET and P3MEEET respectively.

Electrochemical quartz crystal microbalance with dissipation monitoring (EQCM-D) was utilised to determine the mass exchange between the electrolyte and the active channel material. Upon the application of a doping potential (matching the magnitude which resulted in maximum OECT transconductance), significant variation in swelling is observed across the series (Fig. 5(c)). The ethyl spacer derivative, P3MEEET, showed a mass uptake which was 12 times that of P3MEET and P3MEEMT, leading to volumetric capacitance values which aligned with those calculated from electrochemical impedance spectra from OECT channels. It was postulated that the inclusion of a progressively extended alkyl spacer led to increased accessibility of the diethylene glycol side chain imparting heightened ionic transport and increased crystallinity for P3MEEET. Importantly, these studies exemplify the inclusion of a hybrid spacer side chain, which may seem like a simple synthetic modification, has a dramatic effect on numerous properties of the OMIEC.

**Fig. 16:**
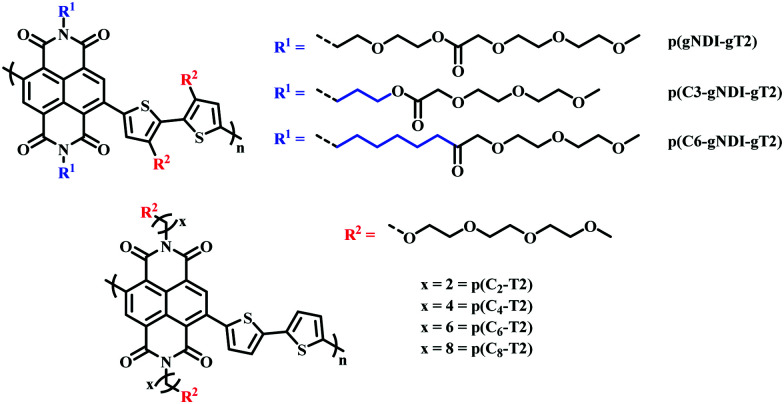
Chemical structures of NDI-T2 derivatives, containing different spacer side chains.^27,90^

Recently, the effect of alkyl spacers on n-type D–A NDI based OMIECs has been investigated.^27,90^ Based on the initial p(gNDI-gT2)^81^ polymer, two alkyl spacer derivatives were synthesised introducing a propyl (C_3_) or hexyl (C_6_) spacer, p(C3-gNDI-gT2) and p(C6-gNDI-gT2), respectively (Fig. 16).^90^ It was proposed that the hybrid alkyl-glycol side chain may protect the polaron, which is delocalised along the polymer backbone, from mobile ions during OECT operation, preventing charge trapping, aiming to increase charge carrier mobility. EQCM-D measurements revealed that both passive and active bias induced (doped) swelling followed the same trend, increasing from p(C6-gNDI-gT2) < p(C3-gNDI-gT2) < p(gNDI-gT2). Demonstrating that decreasing the overall hydrophilic EG density, through the inclusion of hybrid spacer side chains, is an effective synthetic design strategy to modulate the degree of swelling, an important factor which must be considered for high OECT performance.^91^ Similar trends were also observed for both operational stability and overall OECT performance, with both alkyl spacer containing derivatives recording a higher μC* than the solely glycolated derivative p(gNDI-gT2). This study further highlights the importance of side chain engineering, corroborating the previous hybrid side chain studies in confirming that a balanced mix of hydrophilic and hydrophobic side chain density leads to improved OECT operational stability, an essential property for widespread use in bioelectronics.^92^

A similar design strategy was employed by Ohayon *et al.*, presenting a series of NDI D–A polymers, incorporating an alkyl spacer on the NDI unit and polymerising with an unsubstituted bithiophene (T2) donor (p(C_*x*_-T2) series) (Fig. 16), a methoxy decorated bithiophene unit (p(C4-T2-OMe)) and an alkyl spacer containing bithiophene monomer (p(C4-T2-C_*y*_-EG) series), respectively.^27^ The authors found that the electron donating nature of the methoxy groups sufficiently increased the donor ability of the bithiophene unit resulting in ambipolar charge transport, impeding n-type performance. The inclusion of an alkyl spacer in a hybrid alkyl-glycol side chain also resulted in ambipolar operation, as such the authors focus on the p(C_*x*_-T2) series, whereby the unsubstituted bithiophene unit facilitated purely n-type behaviour. OECT mobility increased upon lengthening the alkyl spacer, between the NDI backbone and the EG side chain, peaking for the hexyl spacer (4.74 × 10^−3^ ± 4.31 × 10^−4^ cm^2^ V^−1^ s^−1^) before decreasing for the eight-carbon spacer derivative. The trend in OECT performance was justified by *ex situ* GIWAXS measurements, whereby polymer films were electrochemically reduced and exposed to an aqueous electrolyte solution, mimicking OECT operation. No notable structural changes were observed for the p(C_*x*_-T2) series, compared to their undoped “as cast” pristine state, suggesting that the introduction of an alkyl spacer led to crystalline regions with heightened orientation and stability during OECT operation. In contrast the previously reported glycolated analogue p(gNDI-gT2) displayed significant changes in relative peak intensities under replicated OECT operation conditions, in the absence of any spacer moiety.^81^ The efficacy of alkyl spacer side chains was further bolstered by the state of the art μC* figure of merit recorded for p(C_6_-T2) which at 1.29 ± 0.117 F cm^−1^ V^−1^ s^−1^ is among the highest reported for any n-type active channel OECT material.^93^

These studies demonstrate the power of side chain engineering on the resultant polymer properties and suggest that the inclusion of a hydrophobic alkyl spacer unit between the polymer backbone and a hydrophilic EG side chain can be an effective design strategy to improve adhesion, stability, performance and modulate swelling. However, a careful balance must be reached between the side chain features and performance parameters. Akin to each unique design strategy presented within this review, there is no one-size-fits-all, blanket strategy to produce high performing OMIECs. Nevertheless, a nuanced and systematic approach to chemical design should include the effects of hybrid/spacer side chains as a point of consideration.

### 3.4 Charged side chains

**Fig. 17:**
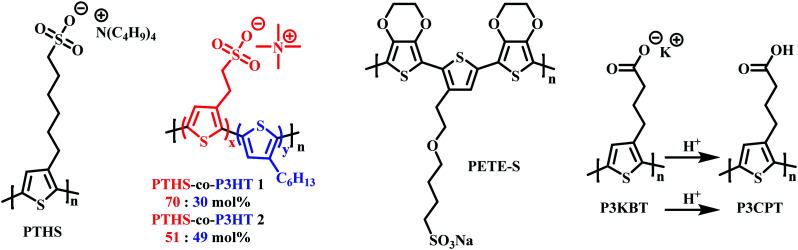
Chemical structures of charged side chain containing OECT active channel layer materials.^96,99,100,102^

As mentioned in Section 2.2, to date the most commonly employed active material within OECTs is PEDOT:PSS owing to the general commercial availability, high conductivity and ease of processing.^45^ Here the positively charged PEDOT backbone is stabilised by the negative PSS^−^ counterion. The widespread availability of PEDOT:PSS has undoubtably bolstered the progress of recent OECT research. However, the limitations related to synthetic polymer modifications render structure–property relationships difficult to elucidate. Multiple studies have postulated the addition of dopants or alternative blends as potential avenues to overcome these limitations.^94,95^ In 2014, Inal *et al.*, presented poly(6(thiophene-3-yl)hexane-1-sulfonate) (PTHS) (Fig. 17) a P3HT analogue, replacing the typical hexyl side chain with a hexanesulfonate side chain affording a conjugated polyelectrolyte with improved ionic conductivity in OECTs.^96^

Indeed, the inclusion of the hydrophilic sulfonate unit improved ionic conductivity by imparting swelling ability, facilitating water and ionic uptake, resulting in a volumetric capacitance of 124 ± 38 F cm^−3^.^97^ High transconductance was also recorded on account of the impressive hole mobility ((1.2 ± 0.5) × 10^−2^ cm^2^ V^−1^ s^−1^) and was much improved over the fully alkylated hydrophobic P3HT.^98^ Following the introduction of PTHS various sulfonated thiophene moieties have been utilised within multiple polythiophene backbones in order to bolster ionic conductivity. In 2019, the THS monomer was copolymerised with 3-hexylthiophene to afford polymers with differing ratios of THS:3HT (Fig. 17).^99^ The authors demonstrated the advantages of including both the 3HT unit which reduced water solubility, hindering delamination, and avoided the need for external crosslinkers with the swellability, ionic conductivity and high C* properties of the THS unit. Similar hole mobilities and volumetric capacitance to that of the aforementioned PTHS were recorded, with OECT operation occurring at a lower threshold voltage with heightened ON/OFF ratios. These studies further highlight the importance of side chain composition, especially the nature and ratio of hydrophilic to hydrophobic side chain density on the resultant ionic conductivity, swelling and general OECT performance. Another example of a sulfonated polymer was presented as an evolvable OECT channel material for neuromorphic applications.^100,101^ Here the hybrid accumulation–depletion mode OECT is formed *in situ via* electropolymerisation of 4-(2-(2,5-bis(2,3-dihydrothieno[3,4-*b*][1,4]dioxin-5-yl) thiophen-3-yl)ethoxy)butane-1-sulfonate (ETE-S), a self-doped conjugated monomer. Upon electropolymerisation, to form PETE-S (Fig. 17), the gate electrode acts as the presynaptic terminal, the polymeric channel controls the synaptic weight, and the drain electrode mimics the postsynaptic terminal, replicating a biological synapse.^100,101^

Reichmanis *et al.*, presented a water-soluble precursor polymer, P3KBT, possessing a charged side chain which could be protonated through acidification to yield the solvent-resistant material poly[3-(4-carboxypropyl)thiophene] (P3CPT) (Fig. 17).^102^ The authors note that the use of carboxylic acid functionalised side chains can act as solubility mediators, whereby the deprotonated, charged, carboxylated salt can be processed from water, allowing facile channel fabrication, whilst post-processing protonation renders the material resilient to delamination improving OECT operational stability. Another important strategy to consider within the OECT side chain toolbox of design principles.

**Fig. 18:**
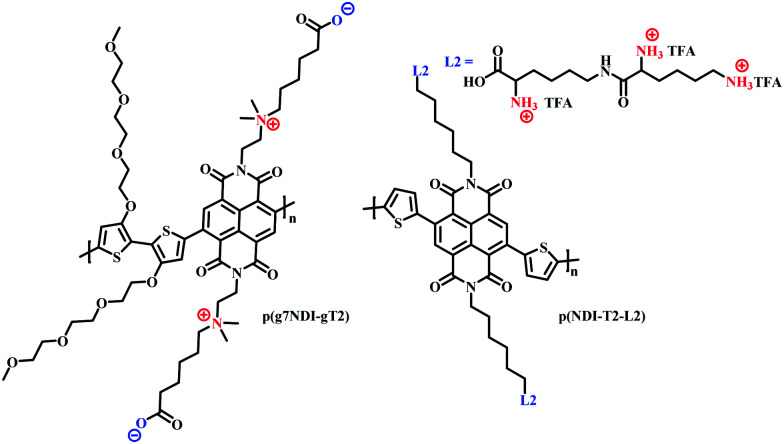
Chemical structures of NDI cored OECT channel materials bearing charged side chains.^85,103^

The use of charged side chains has also been investigated in electron transport (n-type) materials.^85,103^ Moia *et al.*, attached a zwitterionic side chain to an NDI core, copolymerising with a glycolated bithiophene monomer to afford p(g7NDI-gT2) (Fig. 18). The positively charged ammonium ion was synthetically tethered to the NDI core *via* an ethyl spacer, postulating that the negatively charged polymer backbone would be compensated by the opposing charge of the ammonium ion, negating the need for charge compensation *via* mobile cations from the electrolyte. Indeed, the inclusion of the zwitterionic side chain led to enhanced redox reversibility and specific capacity in aqueous electrolytes.

Recently, the first n-type biofunctionalised polymer was reported, tethering a lysine inspired side chain to an NDI core to afford p(NDI-T2-L2) (Fig. 18).^103^ The charged lysine-based side chains enabled electrical communication between the OECT and supported lipid bilayers (SLB), which are a promising platform to study numerous cellular events.^104^ The polarity and surface orientation of the lysine-based side chain allowed for interactions with zwitterionic lipid vesicles, forming SLBs, whilst simultaneously providing the hydrophilicity to afford a volumetric capacitance of 95 F cm^−3^. EQCM-D measurements suggested that the nature of the lysine side chain also limited polymer swelling, with only an 8% increase in original film thickness observed within an aqueous-based salt solution.^103^ This is in contrast to PEDOT:PSS based SLB monitoring OECT devices which show polymer swelling of up to 80%, which could be detrimental to SLB formation.^105,106^ Moreover, this seminal report presented the first report of an n-type OECT capable of both interfacing and monitoring the biomimetic model SLB.

## 4. Conclusions and outlook

In this review, we have summarised the current understanding of the OECT operational mechanisms and specifically, the knowledge of ionic–electronic coupling, which sheds light on the significance of side chain engineering in the active channel layer material. The versatile synthetic toolbox of side chains can be employed to tune OECT properties impacting the material transconductance, morphology, selectivity, sensitivity, and operational stability, and has led to promising OECT device performance.

The composition and nuances of side chains, which have been synthetically tethered to channel materials for OECTs is as vast as the number of backbones which have been studied.^21,93^ Whilst PEDOT:PSS has dominated the field of OECT applications the wealth of examples employing side chain engineering, specifically for OECT active channel materials, is extensive and offers valuable insight into the importance this synthetic tool has. Indeed, the so called “second generation” of semiconducting polymers, namely polythiophene materials, has demonstrated the efficacy of EG based side chains as the current gold standard to produce OMIECs for OECTs.

From the evolving understanding of what determines OECT channel material performance and how side chains influence these metrics, we summarise the findings in the following way: firstly, the hydrophilicity is the most important intrinsic factor that determines the ability of ion uptake, especially in the application of bioelectronics whereby an aqueous environment and ion exchange are essential. Replacing hydrophobic side chains with hydrophilic chains, *e.g.*, EG side chains, is the primary adaptation of the molecular design principles from other thin film application of CPs to meet the working mechanism existing in OECTs. The commercial availability, ability to facilitate ionic transport and the capability to tune polymer swelling are just three reasons why EG based side chains have accelerated to the forefront of molecular design for OECT channel materials. However, these benefits of glycol side chain building blocks may also deter a deeper investigation into side chain engineering. It should not be understated that side chain engineering can become exponentially difficult synthetically, one could envisage the lengthy process needed to synthesis an EG side chain replacement, starting from non-commercially available resources, eventually resulting in a poorer performing channel material which has undoubtedly hindered research in this area. As such, EG side chains might not be the perfect choice for OECT channel materials, however, without significant investment into side chain engineering of novel alternatives EG side chains will continue to be employed for the foreseeable future. Despite this, as exemplified throughout, EG based side chains have undoubtably bolstered the field, improved OECT device performance by orders of magnitude, increased operational stability and have allowed for the swellability of materials to be controlled.^77,79,84,91^

In further examination of how the manipulation of EG side chain percentage, length, and position could influence the polymer performance, the swelling behaviour brought by the engineered hydrophilicity becomes the major parameter to research on. With increasing hydrophilicity in the system, uneven swelling and water retention upon cyclic electrochemical doping processes can indeed permanently disrupt the microstructure permanently and hence weakens material stability and hinders charge carrier mobility.^50,84^ Consequently, one of the major challenges of material design rises from the trade-off between the electronic conductance and ionic conductance, which are opposingly correlated with the swelling behaviour. The introduction of mixed types of side chains into these materials could serve to optimise the charge mobility and the volumetric capacitance simultaneously. In studies examining the potential application of D–A copolymers in OECTs, side chains of different natures could be separately engineered to the donor or acceptor monomers, achieving the goal of controlled swelling^84^ and improved stability.^41,86^

While PEDOT:PSS retains popularity as a widely used OMIEC, featuring the positively charged PEDOT backbone stabilised by the negative PSS^−^ counterion, a number of homogeneous mixed conductors decorated with charged ionic or zwitterionic side chains have been reported. Not only did the introduction of charged side chains improve hydrophilicity of the materials, and consequently, swelling ability and transconductance, but it was also found to improve hole mobility. Furthermore, introducing various ratios of charged and alkyl groups aids to strike the balance between the swelling ability of the mixed conductor and its performance.^96,97^ Last but not least, introduction of zwitterionic side chains was shown to enhance redox reversibility and specific capacity in aqueous electrolytes.^102^ As a result, charged side chains offer tremendous opportunities for fine tuning the hydrophilicity and swellability of a mixed conducting polymer, while maintaining OECT performance.

Utilisation of functionalised side chains afford promising opportunities for the development of novel bioelectronic devices, with tailored selectivity (*e.g.*, sialyllactose and crown ether derivatives). The ability of the functionalised side chains to bind specific analytes (*e.g.*, proteins) paves the way towards wearable sensors and point-of-care evaluation of various viruses, with improved sensitivity and specificity.^107^ However, a trade-off between the hydrophilicity, transconductance, and operational stability must be struck. Hybrid side chains offer the potential to strike a balance between adhesion, stability, performance and swelling and warrant further study.

The prevalence of EG side chains also begs the question of alternative Group 16 (chalcogen) containing side chains such as thioethers which could provide additional stabilising interactions with common polythiophene backbones but come at the unquestionable synthetic cost compared to the commercially available EG side chains. One could also envisage an expansion past EG side chains to more biocompatible units which target specific biological interfaces such as hydrolytically degradable water-soluble side chains.

The wealth of studies summarised above demonstrate the breadth of choices available when choosing which side chain to employ for an OECT channel material. Whilst great strides have been made in terms of processability, modulation of swelling and overall OECT device performance future work will continue to utilise side chain engineering as a major tool to further improve future OECT channel materials. As the field continues to mature more detailed investigations into the crucial role side chain engineering plays in the resultant polymer properties will allow for novel side chain alternatives to be devised and will ultimately lead to further enhancements within the field of OECT channel materials.

## Conflicts of interest

The authors have no conflicts of interest.

## Supplementary Material
